# Adaptive light: a lighting control method aligned with dark adaptation of human vision

**DOI:** 10.1038/s41598-020-68119-7

**Published:** 2020-07-08

**Authors:** Yui Takemura, Masaharu Ito, Yushi Shimizu, Keiko Okano, Toshiyuki Okano

**Affiliations:** 1 Department of Electrical Engineering and Bioscience, Graduate School of Sciences and Engineering, Waseda University, TWIns, Wakamatsucho 2-2, Shinjuku-Ku, Tokyo, 162-8480 Japan; 2 The Smart Life Science Institute, ACROSS, Waseda University, Tokyo, Japan

**Keywords:** Circadian rhythms and sleep, Cognitive neuroscience, Visual system

## Abstract

Light exposure before sleep causes a reduction in the quality and duration of sleep. In order to reduce these detrimental effects of light exposure, it is important to dim the light. However, dimming the light often causes inconvenience and can lower the quality of life (QOL). We therefore aimed to develop a lighting control method for use before going to bed, in which the illuminance of lights can be ramped down with less of a subjective feeling of changes in illuminance. We performed seven experiments in a double-blind, randomized crossover design. In each experiment, we compared two lighting conditions. We examined constant illuminance, linear dimming, and three monophasic and three biphasic exponential dimming, to explore the fast and slow increases in visibility that reflect the dark adaptation of cone and rod photoreceptors in the retina, respectively. Finally, we developed a biphasic exponential dimming method termed Adaptive Light 1.0. Adaptive Light 1.0 significantly prevented the misidentification seen in constant light and effectively suppressed perceptions of the illuminance change. This novel lighting method will help to develop new intelligent lighting instruments that reduce the negative effect of light on sleep and also lower energy consumption.

## Introduction

Poor sleep quality has become a social issue, and a lack of sleep increases the risk of high blood pressure^1^, diabetes^2^, and depression^3^. Insufficient sleep results in excessive activation of the amygdala, and amplifies negative emotions such as anxiety and confusion^4,5^. The causes of insufficient sleep are thought to include exposure to artificial light at night, in addition to psychological factors such as stress^6^ and pharmacological factors such as alcohol and caffeine^7,8^. Light stimulation during the night induces the suppression of melatonin secretion^9^ as well as phase delay of the circadian clock^10^, both of which depend on illuminance of environmental lights^11,12^. These photic effects are mediated by opsins in cone and rod photoreceptor cells, and melanopsin in intrinsically photosensitive retinal ganglion cells (ipRGCs) in the retina^13^. Of note, melanopsin is activated by blue light^14,15^ that is often contained in screen displays of smartphones and personal computers, and hence their use before the onset of sleep is thought to interfere with good quality sleep. In fact, there is a positive correlation between sleep disorders and use of smartphones after ‘lights-off’ before sleep, and use of a mobile tablet device before sleep onset has negative effects on both sleep quality and circadian physiology^16^. Thus, the environmental light condition considerably affects human circadian clocks and drowsiness.

The economic losses caused by inadequate sleep due to detrimental effects on health, overall well-being and productivity, are estimated to be $280–$411 billion in the United States, and $88–$138 billion in Japan^17^. Lowering the light intensity before sleep helps to maintain regular 24 h oscillation of the circadian clock, and would reduce these economic losses. In addition, lowering of the light intensity during night-time directly leads to reduction of energy consumption. Most of the power for lighting is provided by fossil energy sources such as oil and natural gas, but they will be depleted by the end of this century^18^. Therefore, reduction of night lighting may be important also from the point of view of the Sustainable Development Goals (SDGs). However, dark housing environments degrade the quality of life (QOL) and possibly increase the risk of home accidents^19^, so it is important to lower the illuminance of light without lowering the QOL.

In this study, we aimed to develop a novel method to control environmental light conditions before sleep, during which the illuminance of light is reduced so that the change in environmental lighting conditions is not perceived. For this purpose, the biphasic increase in visibility during dark adaptation^20,21^ was taken into account, and here we propose a novel lighting control method. This method, termed Adaptive Light 1.0 (AL1.0), is composed of the first and second dimming steps considering cone and rod adaptations, respectively.

## Results

### Experimental setup, determination of the examination procedures, and experimental design

**Figure 1:**
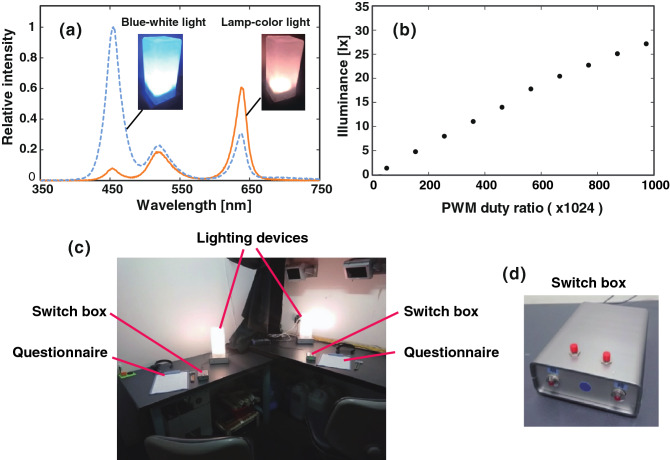
Lighting device and experimental environment. (**a**) Emission spectra of lamp-colour light and blue-white light were measured using a PMA-11 Multi-channel spectrometer (Hamamatsu Photonics, Hamamatsu, Shizuoka, Japan). (**b**) The linearity between the PWM ratio and the illuminance at 100 cm from the lighting device (lamp-colour light, measured by the illuminance meter LUX HiTESTER 3423, Hioki, Nagano, Japan). (**c**) The examination rooms. The examination rooms were compartments in a large, dark room, separated by black curtains. When two subjects participated in the experiment, a black curtain was used to separate the compartment into two compartments, where the light conditions were controlled independently. (**d**) A switch box with “brightened” and “darkened” buttons to ascertain the timing of when subjects perceived the change in illuminance. When pressed twice or more within one second, it was treated as one time. The timing when the switch was pressed was recorded using a voltage logger (LR8431, Hioki).

**Figure 2:**
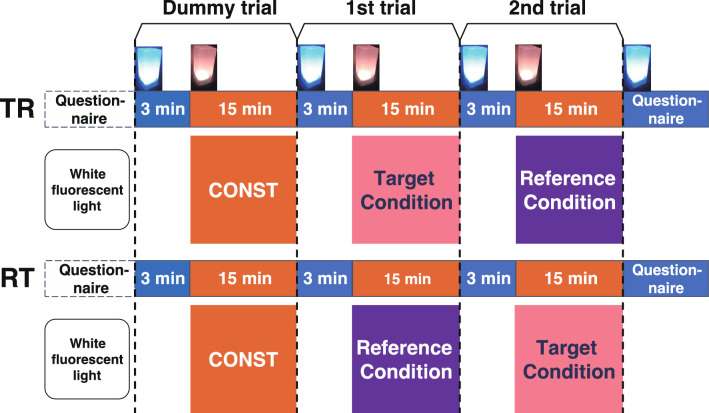
Experimental protocol: double-blind, randomized, crossover design. The subjects experienced three trials, the initial dummy trial (CONST; light with constant illuminance) and two test trials, each comprised of 3 min of conditioning light illumination and 15 min of lamp-colour light illumination. Half of the subjects experienced the target and reference conditions of light illumination in this order (TR pattern), and the other half of the subjects had the reverse order (RT pattern). The assignment of subjects to these patterns was randomly determined, and double-blinded.

During the course of early preliminary experiments, we improved the experimental setup and protocol, and finally determined the experimental environments (Fig. 1) and protocol (Fig. 2). We adopted the protocol in which the results from the latter two of three trials were compared (Fig. 2), because the initial trial (termed "dummy trial") was necessary for the subject to familiarize and adapt to the experimental condition. In each trial, the subject was exposed to 15 min lamp-colour light after 3 min of blue-white light (termed conditioning light) to minimize the effects of the previous trial.

**Figure 3:**
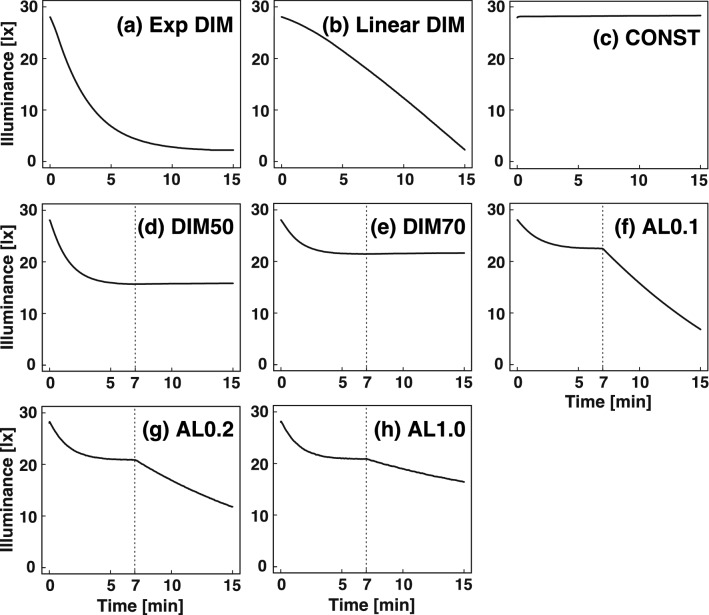
Lighting profiles used in this study. (**a**) Exp DIM; dimming exponentially from 28 to 2 lx over 15 min. (**b**) Linear DIM; dimming linearly from 28 to 2 lx over 15 min. (**c**) CONST; constant illuminance at 28 lx for 15 min. (**d**) DIM50; dimming exponentially to 50% of the first illuminance over the first 7 min, followed by constant illuminance for 8 min at the end of the trial. (**e**) DIM70; dimming exponentially to 70% of the first illuminance over the first 7 min followed by constant illuminance for 8 min at the end of the trial. (**f**) Adaptive Light 0.1; dimming exponentially to 70% over the first 7 min followed by exponential dimming to 20.4% over the last 8 min at the end of the trial. (**g**) Adaptive Light 0.2; dimming exponentially to 70% over the first 7 min followed by exponential dimming to 37.7% over the last 8 min at the end of the trial. (**h**) Adaptive Light 1.0; exponential dimming to 70% over the first 7 min followed by exponential dimming to 53.9% over the last 8 min at the end of the trial. The horizontal axis indicates the time after switching from conditioning light to lamp-colour testing light. The dotted lines indicate 7 min from the switching. The vertical axis indicates illuminance of the testing light at a point 100 cm from the lighting device. A photodiode (S9648, Hamamatsu photonics), calibrated with the illuminance meter (LUX HiTESTER 3423, Hioki), was used for the measurement.

The human dark adaptation process is divided into two phases; a fast cone adaptation that occurs just after the transition from light to dark, and saturates within 4–12 min, and the subsequent slow rod adaptation that takes approximately 30 min^20,21^. Considering this, we aimed to determine the biphasic dimming curve by three steps: (i) Determination of the appropriate curve and threshold illuminance for dimming (Experiment 1); (ii) Determination of the first-step dimming curve, relating to the dark adaptation of cones, by comparing DIM50 (Fig. 3d) or DIM70 (Fig. 3e) with CONST (Fig. 3c; Experiments 2-1 and 2-2); and (iii) Determination of the second-step dimming curve, relating to the dark adaptation of rods, by comparing biphasic curves (termed "Adaptive Lights"; AL0.1, AL0.2 and AL1.0; Fig. 3f–h) with DIM70 (Fig. 3e; Experiments 3-1, 3-2 and 3-3) or CONST (Experiment 3-4). The two conditions compared in each crossover experiment were termed ‘target’ and ‘reference’ conditions, for convenience (Fig. 2, Table 1). The age and gender of subjects are shown in Table 2.

**Table 1:** Target and reference conditions used in each random crossover experiment.

Experiment	Target condition	Reference condition
1	Exponential DIM (Exp DIM)	Linear DIM
2-1	DIM50	CONST
2-2	DIM70	CONST
3-1	Adaptive light 0.1 (AL0.1)	DIM70
3-2	Adaptive light 0.2 (AL0.2)	DIM70
3-3	Adaptive light 1.0 (AL1.0)	DIM70
3-4	Adaptive light 1.0 (AL1.0)	CONST

**Table 2:** Subject information.

Experiment	Full age (ave. ± SD)	M:F	Participants
1	20.7 ± 1.75	16:14	30
2-1	21.1 ± 2.90	18:12	30
2-2	20.4 ± 2.14	18:12	30
3-1	19.9 ± 4.06	21:9	30
3-2	20.0 ± 1.68	18:12	30
3-3	20.6 ± 1.76	44:36	80
3-4	22.1 ± 1.80	27:13	40

### Dimming curve and threshold illuminance for the perception of darkening

#### Experiment 1: Exp DIM vs linear DIM

Exponential dimming (Exp DIM; exponential dimming from 28 to 2 lx in 15 min, Fig. 3a; luminosity of the light is indicated by illuminance at 100 cm from the lighting device) and linear dimming (Linear DIM; linear dimming from 28 to 2 lx in 15 min; Fig. 3b) were compared to determine which dimming curve would tend to be perceived as darkened. In each trial, the intensity of the lamp-colour light was ramped down exponentially or linearly, taking 15 min after the light was switched from the conditioning light (3 min). The intensity of the blue component in the conditioning light was much stronger than that of lamp-colour light (Fig. 1a), although the luminosity of lamp-colour light at the start of the measurement (just after the change in the colour of light) was the same as the conditioning light (28 lx). Thus, the increase in visibility occurred during the 15 min of measurement.

**Figure 4:**
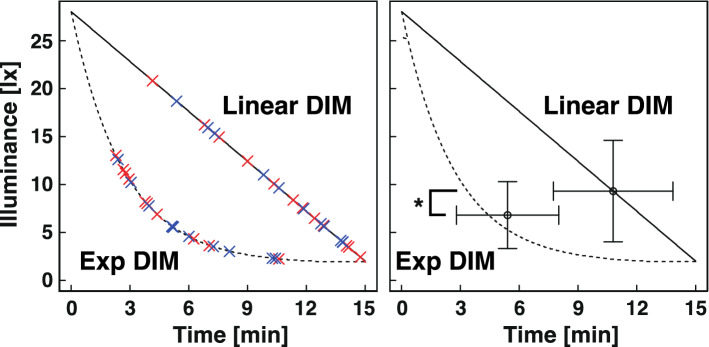
Comparison between exponential and linear dimmings by timings of perception of illuminance changes (Experiment 1). (Left) The timing when subjects perceived “The light was darker than that at Time zero” was plotted on the calculated light intensity curves. Blue and red crosses indicate TR pattern (Exp DIM–Linear DIM) and RT pattern (Linear DIM–Exp DIM), respectively. (Right) The threshold illuminance of perception of illuminance changes calculated from the average and standard deviation of the timings from individual data are shown in the left panel. *p < 0.05 (2 paired t-test).

In this experiment, the subject was guided to operate the switch box (Fig. 1d) only once when he/she felt that the light was definitely "darkened" (decreased illuminance) compared to the start of the illumination (Fig. 4). The average threshold for illuminance (the point at which the subject reported the illuminance to be lower than the initial illuminance) in Exp DIM (6.80 ± 3.49 lx) was significantly lower than that in Linear DIM (9.30 ± 5.29 lx; *p* = 0.0137; Fig. 4, right panel). Based on this result, we concluded that dimming with exponential curves is more suitable for our purpose than linear dimming. There was no significant difference between the threshold illuminances in the Target-Reference (TR) pattern (blue crosses in Fig. 4, left panel) and Reference-Target (RT) pattern (red crosses in Fig. 4, left panel).

The result of the questionnaires indicated that KSS-J relaxation after Exp DIM was significantly higher than that after Linear DIM (6.63 vs 6.03, *p* = 0.04; Table 3). There was no significant difference in electroencephalogram (EEG) data between the two test conditions (Supplementary Table S2).

**Table 3:** Summary of questionnaire answers in Experiment 1 (target condition vs reference condition).

Exp	Target condition Reference condition	Most sleepy^#^	Most relax^#^	Perception of illuminance change^#^	KSS^##^		VAS^##^	
					Sleepiness	Relax	Sleepiness	Relax
1	Exp DIM	40.0 (12, 6, 6)	46.7 (14, 7, 7)	70.0 (21, 10, 11)	5.27 ± 1.95	6.63 ± 1.60*	50.47 ± 20.55	67.57 ± 20.13
	Linear DIM	36.7 (11, 5, 6)	36.7 (11, 6, 5)	73.3 (22, 10, 12)	5.50 ± 1.69	6.03 ± 1.52	52.30 ± 16.10	61.87 ± 18.15

*p < 0.05 (paired *t*-test).

^#^Percentage (Total, TR pattern, RT pattern).

^##^Average ± SD.

### Determination of the first-step dimming curve considering the dark adaptation of cones

#### Experiment 2-1: DIM50 vs CONST

When the light is dimmed according to an appropriate curve that considers the dark adaptation of cones, change of the illuminance may be nearly unrecognized until the completion of cone adaptation. Thus, we first designed DIM50 (exponential dimming to 50% of the initial illuminance in the cone adaptation period [cone adaptation period] followed by constant illuminance in the last 8 min [rod adaptation period]; Fig. 3d) taking into consideration the physiological curve of dark adaptation (see “Methods” section). In this study, the cone adaptation period was fixed at 7 min as the geometric mean of the range of reported values (4–12 min)^20,21^.

**Figure 5:**
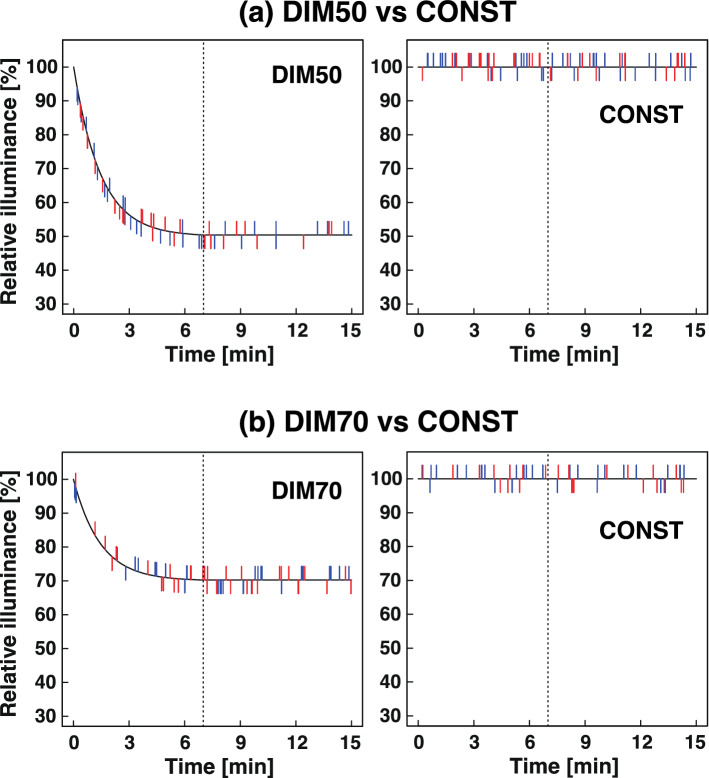
Comparisons between exponential dimmings and constant light by timings of perception of illuminance changes (Experiments 2-1 and 2-2). (**a**) Comparison between DIM50 and CONST. (**b**) Comparison between DIM70 and CONST. The black curves and lines indicate illuminance during exponential dimmings (DIM50 or DIM70) and CONST. The short coloured lines extending upwards from the curve or line indicate the timing when subjects perceived “brightened” conditions, and those extending down indicate the timing when subjects perceived “darkened” conditions. The blue and red lines are data from the TR pattern (DIM50/70–CONST) and RT pattern (CONST–DIM50/70), respectively. The dotted lines indicate 7 min after switching from conditioning light (3 min) to lamp-colour light (15 min).

Comparison of DIM50 with CONST (constant illuminance for 15 min; Fig. 3c) showed that the frequency of “brightened” (increased illuminance) perception in DIM50 was significantly lower than that in CONST in the cone adaptation period (0.40 vs 0.90, 12 vs 27 counts, *p* = 0.002; Table 4, Fig. 5a) and the rod adaptation period (0.37 vs 0.77, 11 vs 23 counts, *p* = 0.008; Table 4, Fig. 5a). This misidentification in CONST may be caused by an increase in visibility during dark adaptation, and the misidentification is likely suppressed in DIM50. On the other hand, the frequency of “darkened” perception in DIM50 was significantly higher than that in CONST in the cone adaptation period (0.90 vs 0.30, 27 vs 9 counts, *p* < 0.001; Table 4, Fig. 5a).

**Table 4:** Frequency of the perception of changes in illuminance.

Exp	Target condition Reference condition	Cone adaptation period (0–7 min)				Rod adaptation period (7–15 min)			
		Brightened		Darkened		Brightened		Darkened	
		Freq.^$^	n^#^	Freq.^$^	n^#^	Freq.^$^	n^#^	Freq.^$^	n^#^
2-1	DIM50	0.40	12 (6, 6)**	0.90	27 (16, 11)***	0.37	11 (6, 5)**	0.30	9 (3, 6)
	CONST	0.90	27 (14, 13)	0.30	9 (5, 4)	0.77	23 (15, 8)	0.47	14 (8, 6)
2-2	DIM70	0.53	16 (8, 8)	0.33	10 (5, 5)	0.67	20 (11, 9)	0.60	18 (8, 10)
	CONST	0.67	20 (13, 7)	0.20	6 (3, 3)	0.50	15 (9, 6)	0.40	12 (5, 7)
3-1	AL0.1	0.30	9 (4, 5)	0.23	7 (5, 2)	0.17	5 (4, 1)	0.93	28 (14, 14)***
	DIM70	0.23	7 (4, 3)	0.17	5 (2, 3)	0.33	10 (4, 6)	0.17	5 (3, 2)
3-2	AL0.2	0.47	14 (9, 5)	0.60	18 (9, 9)*	0.20	6 (4, 2)**	0.53	16 (6, 10)**
	DIM70	0.40	12 (10, 2)	0.30	9 (5, 4)	0.50	15 (9, 6)	0.23	7 (4, 3)
3-3	AL1.0	0.31	25 (12, 13)	0.31	25 (10, 15)	0.36	29 (14, 15)	0.11	9 (5, 4)*
	DIM70	0.38	30 (8, 22)	0.26	21 (6, 15)	0.43	34 (16, 18)	0.23	18 (8, 10)
3-4	AL1.0	0.18	7 (3, 4)***	0.33	13 (9, 4)	0.23	9 (7, 2)*	0.20	8 (4, 4)
	CONST	0.50	20 (9, 11)	0.20	8 (6, 2)	0.40	16 (10, 6)	0.20	8 (4, 4)

^$^Counts (n) per number of subjects.

^#^Total counts (counts in TR pattern, counts in RT pattern).

*p < 0.05; **p < 0.01; ***p < 0.001 (Goodness of fit test for the Poisson distribution).

There was no significant difference in the results of the questionnaire and EEG between the two test conditions (Table 5, Supplementary Tables S1, S2).

**Table 5:** Summary of questionnaire answers in Experiments from 2-1 to 3-4 (target condition vs reference condition).

Exp	Target condition Reference condition	Most sleepy^#^	Most relax^#^	Perception of illuminance change^#^	KSS^##^		VAS^##^	
					Sleepiness	Relax	Sleepiness	Relax
2-1	DIM50	43.3 (13, 7, 6)	36.7 (11, 4, 7)	66.7 (20, 12, 8)	5.20 ± 2.12	6.76 ± 1.59	53.03 ± 22.16	68.63 ± 17.13
	CONST	43.3 (13, 5, 8)	43.3 (13, 5, 8)	53.3 (16, 8, 8)	5.33 ± 2.64	6.48 ± 1.63	54.63 ± 27.11	67.33 ± 21.72
2-2	DIM70	36.7 (11, 8, 3)	40.0 (12, 5, 7)	73.3 (22, 13, 9)	5.87 ± 1.98	6.72 ± 1.20	60.67 ± 22.12	69.37 ± 17.70
	CONST	40.0 (12, 5, 7)	36.7 (11, 5, 6)	60.0 (18, 11, 7)	5.27 ± 2.13	6.48 ± 1.40	58.43 ± 21.89	65.83 ± 19.28
3-1	AL0.1	36.7 (11, 6, 5)	50.0 (15, 4, 11)	70.0 (21, 12, 9)^†^	5.53 ± 2.14	6.80 ± 1.38	56.80 ± 21.57	68.73 ± 16.11
	DIM70	43.3 (13, 5, 8)	26.7 (8, 4, 4)	33.3 (10, 5, 5)	5.87 ± 1.96	6.43 ± 1.31	58.37 ± 22.16	66.70 ± 17.07
3-2	AL0.2	40.0 (12, 7, 5)	40.0 (12, 4, 8)	43.3 (13, 7, 6)	5.53 ± 2.17	6.50 ± 1.41	59.83 ± 24.36	67.53 ± 16.74
	DIM70	30.0 (9, 4, 5)	46.7 (14, 9, 5)	30.0 (10, 5, 5)	5.33 ± 2.27	6.40 ± 1.33	54.43 ± 26.23	65.03 ± 19.87
3-3	AL1.0	30.0 (24, 14, 10)	42.5 (34, 17, 17)	45.0 (36, 21, 15)	5.18 ± 1.99	6.80 ± 1.24	51.15 ± 21.93	67.35 ± 17.27
	DIM70	42.5 (34, 17, 17)	38.8 (31, 17, 14)	42.5 (34, 19, 15)	5.51 ± 2.12	6.89 ± 1.29	55.65 ± 22.24	68.58 ± 19.01
3-4	AL1.0	32.5 (13, 8, 5)	37.5(15, 5, 10)	25.0 (10, 6, 4)	5.40 ± 1.93	6.75 ± 1.56	56.35 ± 21.74	65.98 ± 19.75
	CONST	37.5 (15, 5, 10)	32.5 (13, 6, 7)	32.5 (13, 7, 6)	5.25 ± 2.40	6.53 ± 1.55	51.75 ± 25.71	63.88 ± 19.38

^†^*p* < 0.01 (Significant difference in ratios).

^#^Percentage (Total, TR pattern, RT pattern).

^##^Average ± SD.

#### Experiment 2-2: DIM70 vs CONST

The result of Experiment 2-1 suggested that DIM50 might not be more comfortable than CONST. Therefore, we reduced dimming range from 50 to 30%, in order to decrease the “darkened” perception. DIM70 (exponential dimming to 70% of the initial illuminance in the cone adaptation period followed by constant illuminance in the rod adaptation period; Fig. 3e) was designed and compared with CONST (Fig. 3c).

It is noteworthy that there was no significant difference in the frequency of “darkened” perception in the cone adaptation period (dimming phase in DIM70) between the two test conditions (Table 4, Fig. 5b), suggesting that the significant difference between DIM50 and CONST disappeared when DIM50 was changed to DIM70. No differences were observed in the "darkened" perception of rod adaptation period or "brightened" perception of both rod and cone adaptation periods.

There was no significant difference in the results of questionnaire and EEG between the two test conditions (Table 5, Supplementary Tables S1, S2).

### Determination of the second-step dimming curve considering the dark adaptation of rods

#### Experiment 3-1: adaptive light 0.1 (AL0.1) vs DIM70

Next, we added the second dimming phase, considering the adaptation of rods in the rod adaptation period (7–15 min), expecting that such dimming might decrease the misidentification of “brightened” perception in this period. At first, we designed Adaptive Light 0.1 (AL0.1, defined as exponential dimming to 70% of the initial illuminance in the cone adaptation period followed by exponential dimming to 20.4% of the initial illuminance in the rod adaptation period; Fig. 3f), and compared it with DIM70 (Fig. 3e).

**Figure 6:**
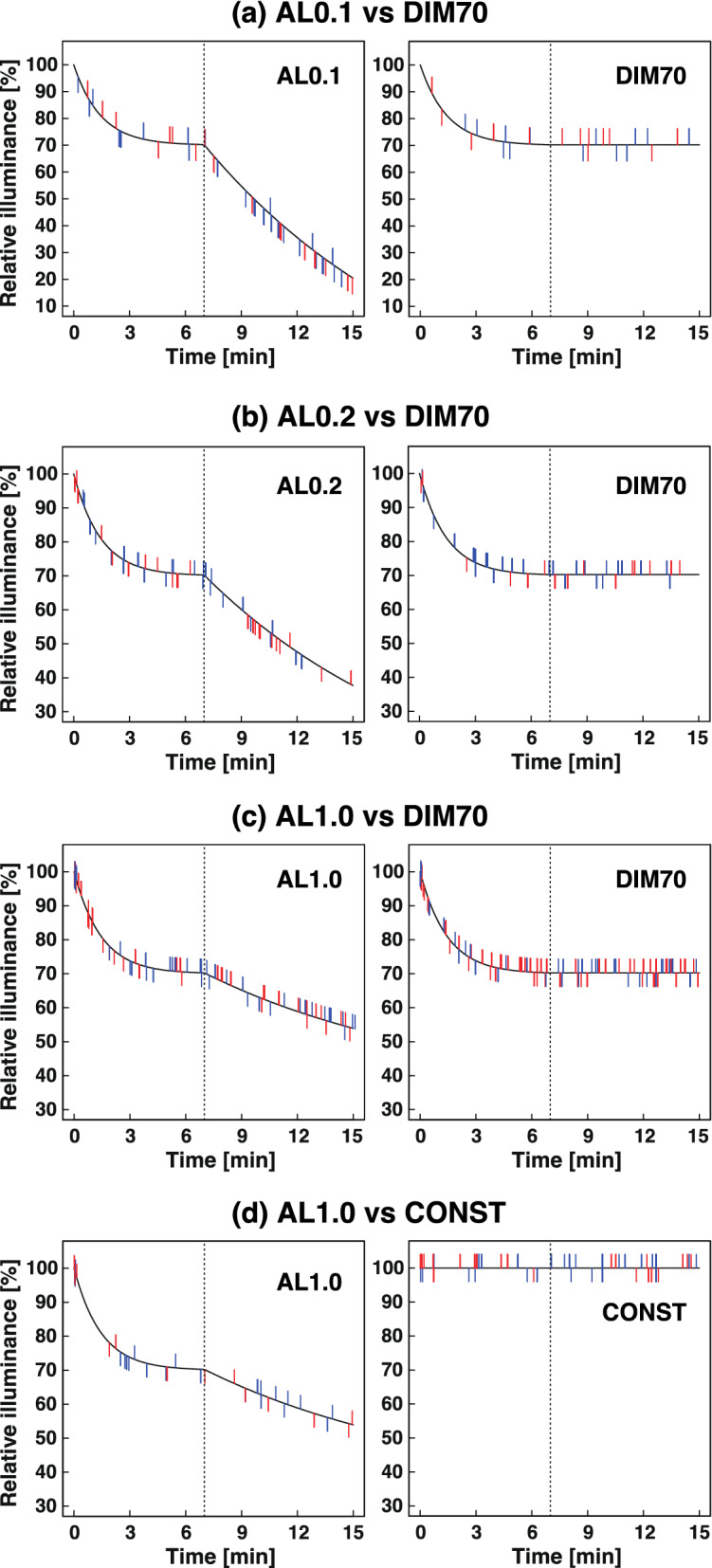
Comparisons between Adaptive Lights (ALs) and exponential dimming (DIM 70) or constant light (CONST) in terms of the timings of perception of illuminance changes (Experiments 3-1 to 3-4). (**a**–**c**) Comparisons between Adaptive Lights ((**a**) AL0.1; (**b**) AL0.2; (**c**) AL1.0) and DIM70. (**d**) Comparison between AL1.0 and CONST. The black curves and lines indicate temporal changes in the illuminance. The short coloured lines extending upward from the curve or line indicate the timing when subjects perceived “brightened” conditions, and those extending down indicate the timing when subjects perceived “darkened” conditions. The blue and red lines are data from the TR and RT patterns, respectively. The dotted lines indicate 7 min after switching from conditioning light (3 min) to lamp-colour light (15 min).

The frequency of “darkened” perception in AL0.1 was significantly higher than that in DIM70 in the rod adaptation period (0.93 vs 0.17, 28 vs 5 counts, *p* < 0.001; Table 4, Fig. 6a). When we compared the results according to the order of trials, the second trial significantly increased the degree of relaxation in the questionnaire, relative to the first trial (VAS, *p* = 0.03; Supplementary Table S1).

In the questionnaire, more subjects reported perceiving the light change in AL0.1 than DIM70 (70.0% vs 33.3%, *p* = 0.005; Table 5). There was no significant difference in EEG data between the two test conditions (Supplementary Table S2).

#### Experiment 3-2: adaptive light 0.2 (AL0.2) vs DIM70

Then, we designed AL0.2 (exponential dimming to 70% in the cone adaptation period followed by exponential dimming to 37.7% of the initial illuminance in the rod adaptation period; Fig. 3g) in order to decrease the dimming range in the rod adaptation period. Comparison of AL0.2 with DIM70 (Fig. 3e) showed, however, that the frequency of “darkened” perception in AL0.2 was still significantly higher than that in DIM70, in both the cone adaptation period (0.60 vs 0.30, 18 vs 9 counts, *p* = 0.015; Table 4, Fig. 6b) and the rod adaptation period (0.53 vs 0.23, 16 vs 7 counts, *p* = 0.0099; Table 4, Fig. 6b). On the other hand, the frequency of “brightened” perception in AL0.2 was significantly lower than that in DIM70 in the rod adaptation period (0.20 vs 0.50, 6 vs 15 counts, *p* = 0.008; Table 4, Fig. 6b). There were no differences in the "brightened" perception in the cone adaptation period.

There was no significant difference in the results of questionnaire and EEG between the two test conditions (Table 5, Supplementary Tables S1, S2). When we compared the results of the questionnaire according to the order of trials, the first trial significantly increased sleepiness compared to the second trial (KSS, *p* = 0.03; Supplementary Table S1).

#### Experiment 3-3: adaptive light 1.0 (AL1.0) vs DIM70

The results of Experiment 3-2 suggested that dimming during the rod adaptation period in AL0.2 remained too fast for our purpose, and thus we further designed AL1.0 (exponential dimming to 70% in the cone adaptation period, followed by exponential dimming to 53.9% of the initial illuminance in the rod adaptation period; Fig. 3h) to be compared with DIM70 (Fig. 3e).

Interestingly, the frequency of “darkened” perception in AL1.0 was reversed, being significantly lower than that in DIM70 in the rod adaptation period (0.11 vs 0.23, 9 vs 18 counts, *p* = 0.015; Table 4, Fig. 6c). No difference was observed in the "darkened" perception of the cone adaptation period. Furthermore, no significant difference was observed in the frequency of “brightened” perception between AL1.0 and DIM70 in both the rod and cone adaptation periods, despite increasing the number participants in this experiment to 80 subjects. There was no significant difference in questionnaire and EEG data between the two test conditions (Table 5, Supplementary Tables S1, S2).

#### Experiment 3-4: adaptive light 1.0 (AL1.0) vs CONST

Results of experiment 3-3 suggested that AL1.0 compensates well for the dark adaptation, so we further evaluated AL1.0 (Fig. 3h) by comparing it with CONST (Fig. 3c).

The frequency of “brightened” perception in AL1.0 was significantly lower than that in CONST in both the cone adaptation period (0.18 vs 0.50, 7 vs 20 counts, *p* < 0.001; Table 4, Fig. 6d) and the rod adaptation period (0.23 vs 0.40, 9 vs 16 counts, *p* = 0.043; Table 4, Fig. 6d). On the other hand, there was no significant difference in the frequency of “darkened” perception between the two test conditions.

The questionnaire results indicated that the first trial significantly increased sleepiness when compared to the second trial (KSS, *p* = 0.004; VAS, *p* = 0.02; Supplementary Table S1). There was no significant difference in the results of questionnaire and EEG between the two test conditions (Table 5, Supplementary Tables S1, S2).

## Discussion

We compared 2 out of 8 lighting conditions (Fig. 3) in 7 experiments, each of which was performed in a double-blind, randomized crossover design. We found many significant differences in the perception of change in illuminance, which were recorded using a switch box (Table 4). Throughout these experiments, we developed Adaptive Light 1.0 (AL1.0) as a novel dimming method, while EEG and KSS-J/VAS in the questionnaire failed to detect a significant difference in the experiments, with the exception of Experiment 1 (Tables 3, 5, Supplementary Table S2). In Experiment 1, KSS-J relaxation in Linear DIM (6.03 ± 1.52, Table 3) was significantly lower than that reported with Exp DIM (6.63 ± 1.60, Table 3). This may be due to the difference in the total amount of light exposure, which was lower in Exp DIM than Linear DIM. Alternatively, Linear DIM has an excitatory effect compared to Exp DIM. The result of Experiment 1 suggests that it is harder to perceive changes in the light intensity with exponential dimming, compared to linear dimming (Fig. 4). This is likely due to the fact that human dark adaptation occurs exponentially^20,21^ (Supplementary Fig. S1) and that the quantity of visual perception is likely to be proportional to the logarithm of the quantity of light stimuli, according to the Weber–Fechner law.

Subjects sometimes misidentified the light illuminance as brightened in CONST and DIM50, yet no increase in the illuminance occurred under all the examined light conditions (upward short lines in Figs. 5, 6). These misidentifications may be attributed to increased visibility by the pupillary opening that may be triggered by decreases in blue light upon switching from the conditioning light to the lamp-colour light^22^. Additionally, spontaneous changes in visual sensitivity likely contributed to the misidentification, because “darkened” misidentifications also occurred in the CONST trials (Table 4, Figs. 5, 6d).

When we compared AL1.0 with CONST (Experiment 3-4), the frequency of “brightened” perception in AL1.0 was significantly lower than that in CONST (Table 4, Fig. 6d), and there was no significant difference in the frequency of “darkened” perception between the two test conditions. Based on these results, we consider AL1.0 to be a light control method that compensates for the increase in visibility and suppresses the perception of the change in illuminance, with adjustment to the biphasic increases in visibility^20,21^. In the future, real-time measurements of visibility during the change in light intensity would help to improve AL1.0, with better compensation of visibility changes. In such improvements, we would need to pay attention to the intensity of the conditioning light, because the dark adaptation profile varies depending on the adaptation light^20,21^.

In comparing DIM70 and AL1.0, a light control method optimized in this study, the frequency of “darkened” perception in AL1.0 was lower than that in DIM70 during the last 8 min (0.11 vs 0.23; Experiment 3-3, Table 4, downward short lines in Fig. 6c), although the illuminance in the last 8 min was higher in DIM70 (Fig. 3e) than AL1.0 (Fig. 3h). This apparent discrepancy could be mechanistically explained by the higher illuminance in DIM70 in the last 8 min potentially inducing pupil contraction.

Application of AL1.0 before sleeping would enable a decrease in the amount of light exposure, without discomfort. Artificial illuminance before sleeping suppresses the secretion of melatonin^11^ and induces the phase regression of the circadian rhythms^12^. Therefore, the application of AL1.0 at night will lead to increased melatonin secretion and hence keep the internal circadian clock healthy. In the present study, we performed the experiments between 18:00 and 20:00, and it is earlier than sleeping times of the subjects. We used this schedule in order to exclude possible effects by differences in individual sleeping times, but it is interesting to investigate how the experimental schedule affect the result. Also, it would be important to test whether AL1.0 has any effects on mood/cognition-related functions such as depression and attention. These possibilities may be examined by both clinical and social applications.

Our results indicate a way to reduce the negative effect of light on sleep, and also to reduce energy consumption. The proportion of electricity consumption attributed to lighting devices is approximately 13.4% of domestic electricity consumption^23^. Social implementation of AL1.0 would contribute to saving fossil fuels for thermal power generation, and also extend the time period before their exhaustion (which, at the end of 2017, is 50.2 years for oil and 52.6 years for natural gas)^18^.

## Methods

### Study design, setting and participants

A cross-over trial was conducted at Waseda University between July 2017 and February 2019. Waseda University is located in Tokyo of Japan and has approximately 52,000 of students. Students selected for this study (n = 270) were drawn from 13 faculties and had to meet the following inclusion criteria: students of 18–40 years old who were native speakers of Japanese. We excluded students who had been overseas within the past week, night workers, drug users, unwell persons, who had awareness of color blindness, pregnant women, and women of childbearing potential due to possible changes in sleep patterns due to travel, occupational reasons, pharmacologic or physiologic reasons respectively. A sample size of 15 was required to have approximately 80% power to detect large effects (effect size of 0.8) at a significance level of 0.05 in two-tailed paired t-test, and the sample sizes were set over 15 (Table 2). In experiment 3-3, sample size was increased to 80 to detect a smaller effect (effect size of 0.32).

### Equipment

We developed lighting devices that were composed of four three-colour LEDs (OSTCXBC1C1S, Optosupply, Hong Kong) and 1-chip microcontroller PIC16F1939 (Microchip Technology, Chandler, AZ, USA), which were covered with a glass block of a frosty block lamp (AW-0332, ARTWORK STUDIO, Kobe, Japan). The LEDs were controlled by a 10-bit pulse width modulation (PWM; Fig. 1b). We used statistical software R version 3.5.0^25^ for calculation of the light pattern, and MPLAB X IDE version 5.05 (Microchip Technology, https://www.microchip.com/mplab/mplab-x-ide) for writing to the microcomputer. We developed switch boxes with “brightened” and “darkened” buttons in order to record the timing of when the subjects perceived the change in illuminance (Fig. 1d).

### Design of dimming curves

Light adaptation is a biphasic process; cone and rod adaptation. Cone adaptation is completed in approximately 4–12 min, and the visual sensitivity is increased by 10–100 times, depending on the adapting intensity^20,21^. After that, the rod adaptation is completed in approximately half an hour, and the visual sensitivity is further increased by about 10,000 times (the black circles in Supplementary Fig. S3). According to Samii and O'Brien^26^, we estimated the time course of the increase in visibility due to dark adaptation (the red curve in Supplementary Fig. S3) by a combination of two exponential curves. We considered that the dark adaptation curves (S_cone_(t) and S_rod_(t), see below) may be directly fitted to profiles of exponential dimming curves, because the threshold of perceived change is likely to be proportional to the logarithm of the stimuli (illuminance), according to the Weber–Fechner law. We created dimming curves by using parameters estimated in the course of the fitting, as follows:

$${\text{S}}_{{{\text{cone}}}} \left( {\text{t}} \right) \, = {\text{A }} + \, \left( {{1 } - {\text{ A}}} \right) \, \times \, 0.{996787282}^{{\left( {{\text{t}}/0.{28}} \right)}} \quad (0\,{ \leqq }{\text{t}} < {42}0)$$

where A is destination intensity for t = ∞ (0.5 and 0.7 for DIM50 and DIM70, respectively).

$${\text{S}}_{{{\text{rod}}}} \left( {\text{t}} \right) \, = {\text{B }} + \, \left( {0.{7 } - {\text{ B}}} \right) \, \times \, 0.{999637396}^{{\left( {\left( {{\text{t}} - {42}0} \right)/0.{28}} \right)}} \quad ({42}0\,{ \leqq }{\text{t}}\,{ \leqq }{9}00)$$

where B is destination intensity for t = ∞ (− 0.37, 0, and 0.35 for AL0.1, AL0.2 and AL1.0, respectively).

Exponential dimming curves (DIM50 and DIM70) were created by combining S_cone_(t) (0 ${ \leqq }$ t < 420) and constant illuminance (420 ${ \leqq }$ t). Adaptation Lights were created by combining S_cone_(t) (0 ${ \leqq }$ t < 420) and S_rod_(t) (420 ${ \leqq }$ t).

### Protocol and light exposure procedures

One or two subjects participated in the experiment at a time, between 18:00 and 20:00, in the examination room (Fig. 1c). The subject was entered the examination room (Fig. 1c) at least 30 min before commencing the experiment, and completed a pre-questionnaire under a white fluorescent lamp (260 lx, FHF32EX-N-HX-S, NEC lighting, NEC Corp., Minato City, Tokyo, Japan). Each subject was seated on the chair at a position approximately 100 cm away from each lighting device on the desk. The examination comprised of the dummy trial, to familiarize the subject to the trials, and the first and second trials for comparison (target condition and reference condition in Fig. 2). The three trials were carried out continuously in the same manner, and the subjects were not informed that there would be a dummy trial. Each trial was composed of 3 min of conditioning light (blue-white light; 28 lx, Fig. 1a) and test light [lamp-colour light (Fig. 1a) with constant or temporally changing intensities (Fig. 3)]. The “CONST” pattern (Fig. 3c) was used for the dummy trial. In the first and second trials, the target condition and reference condition (Table 1) were cross-tested: the target condition was followed by the reference condition (TR pattern), or the reference condition was followed by the target condition (RT pattern). The experiment was double-blind so that both the experimenter and subject did not know which of the pattern were tested in the examination as follows: Same number (half of the total number of subjects) of microcomputer chips storing either program for the TR or RT pattern were prepared for the total number of subjects, and used in a randomized order that was determined by the person other than experimenter. In each examination, the experimenter set the microcomputer chip to the lighting device and did not stay in the examination room during the examination.

We conducted 7 different experiments. Experiment 1 determined the appropriate curve and threshold illuminance for dimming, Experiments 2-1 and 2-2 determined the first step dimming curve, Experiments 3-1, 3-2, 3-3 and 3-4 determined the second step dimming curve.

We guided the subject to operate the switch box during the 15 min test light; only once at the one illumination when the subject felt that it was clearly darker compared to the start of illumination (Experiment 1), or every time subjects perceived changes in the light intensity (all experiments except for Experiment 1).

EEG was measured from the difference in skin potential between the forehead and the earlobe using a simple electroencephalograph, MindWave Mobile (NeuroSky, San Jose, CA, USA). Alpha wave per beta wave and “Meditation”, which are generally used as indices of the degree of relaxation, were used as evaluation indices. In cases where the EEG data was not recorded, the other data were independently processed.

After the three light exposures, the subjects completed a questionnaire under conditioning light (Fig. 2). The questionnaire was a Japanese translation of the Karolinska Sleepiness Scale (KSS-J^27^), visual analogue scale (VAS^28^) and the Japanese version of the content shown in Supplementary Method: Questionnaire.

We did not notify the subjects that the initial trial was a dummy trial. We analysed the data obtained in the first and second trials.

### Statistical analysis

In the questionnaire, we asked subjects to indicate the light conditions that made them feel the sleepiest and the most relaxed. We excluded answers that chose the dummy trial, and compared numbers answering the first or second trial using a binomial test (two-sided test, significance level 0.05). For the evaluation of drowsiness and relaxation using the VAS and KSS-J methods, paired *t*-tests (two-sided test, significance level 0.05) were used.

In relation to the EEG, the indices from the target and reference trials were normalized according to the users' guide of NeuroExperimenter (the square root of each wave value divided by the sum of all wave values) and compared using the Wilcoxon rank sum test (two-sided test, 0.05 significance level). Then, the number of participants showing significant differences were compared using a Binomial test (two-sided test, significance level 0.05).

We analysed the timings of "brightened" or "darkened" perception obtained by real-time recording using a switch box as follows: in experiment 1, we compared the illuminance when the subject reported the light to be darkened, using a paired *t*-test (two-sided test, significance level 0.05). In the other experiments, we compared the number of times participants perceived “brightened” and “darkened” environments by using a goodness of fit test for the Poisson distribution (two-sided test, significance level 0.05) in the cone adaptation period (0–7 min) and in the rod adaptation period (7–15 min), separately. Data on subjects whose total number of times pushing the button was outside the range of average ± 2 × standard deviation were excluded as abnormal values.

### Ethics statement

All experiments were carried out in accordance with the Code of Ethics relating to Research with Human Subjects, of the Office of Research Ethics of Waseda University, and were approved by the Review Committee for Research with Human Subjects, Waseda University (Application number: 2017-046). Informed consent for research was obtained from all participants. The safety of the light exposure using in the experiments was confirmed in accordance with Japanese Industrial Standards (Photobiological safety of lamps and lamp systems, JIS 7550)^24^.

## Supplementary information

Supplementary Information

## Data Availability

The data supporting the findings of this study are available within the paper and its Supplementary Information files.
