# Supplementary material for: Adaptive light: a lighting control method aligned with dark adaptation of human vision
Source: Sci Rep. 2020 Jul 8;10:11204. doi: 10.1038/s41598-020-68119-7 (PMC7343865; doi:10.1038/s41598-020-68119-7)
Supplement: Supplementary file 1 — Supplementary Information [file 41598_2020_68119_MOESM1_ESM.docx]

Supplementary Information for

**Adaptive Light: A lighting control method aligned with dark adaptation of human vision**

Yui Takemura^+^, Masaharu Ito^+^, Yushi Shimizu^+^, Keiko Okano^+^, Toshiyuki Okano^+,$,^*

**Affiliation**

^+^Department of Electrical Engineering and Bioscience, Graduate School of Sciences and Engineering, Waseda University, TWIns, Wakamatsucho 2-2, Shinjuku-Ku, Tokyo 162-8480, Japan.

^$^The Smart Life Science Institute, ACROSS, Waseda University.

***Corresponding Author:**[okano@waseda.jp](mailto:okano@waseda.jp)

## Supplementary Tables

**Supplementary Table S1. Questionnaire results sorted by the order of trials.**

| Exp | Target condition Reference condition | Most sleepy# | Most relax# | Perception of illuminance change# |  | KSS## | | |  | VAS## | | |  |
| --- | --- | --- | --- | --- | --- | --- | --- | --- | --- | --- | --- | --- | --- |
|  |  |  |  |  |  | Sleepiness |  | Relax |  | Sleepiness |  | Relax |  |
|  |  |  |  |  |  |  |  |  |  |  |  |  |  |
| 1 | First | 40.0 (12, 6, 6) | 40.0 (12, 7, 5) | 73.3 (22, 10, 12) |  | 5.60 ± 2.04 |  | 6.43 ± 1.65 |  | 53.40 ± 19.77 |  | 64.77 ± 20.30 |  |
|  | Second | 36.7 (11, 5, 6) | 43.3 (13, 6, 7) | 70.0 (21, 10, 11) |  | 5.17 ± 1.55 |  | 6.23 ± 1.52 |  | 49.37 ± 16.86 |  | 64.67 ± 18.41 |  |
|  |  |  |  |  |  |  |  |  |  |  |  |  |  |
| 2-1 | First | 50.0 (15, 7, 8) | 40.0 (12, 4, 8) | 66.7 (20, 12, 8) |  | 5.53 ± 2.29 |  | 6.45 ± 1.30 |  | 56.30 ± 23.12 |  | 67.47 ± 18.34 |  |
|  | Second | 36.7 (11, 5, 6) | 40.0 (12, 5, 7) | 53.3 (16, 8, 8) |  | 5.00 ± 2.46 |  | 6.79 ± 1.86 |  | 51.37 ± 26.10 |  | 68.50 ± 20.72 |  |
|  |  |  |  |  |  |  |  |  |  |  |  |  |  |
| 2-2 | First | 50.0 (15, 8, 7) | 36.7 (11, 5, 6) | 66.7 (20, 13, 7) |  | 6.00 ± 1.97 |  | 6.69 ± 1.21 |  | 63.63 ± 22.47 |  | 69.57 ± 18.22 |  |
|  | Second | 26.7 (8, 5, 3) | 40.0 (12, 5, 7) | 66.7 (20, 11, 9) |  | 5.13 ± 2.09 |  | 6.52 ± 1.40 |  | 55.47 ± 20.80 |  | 65.63 ± 18.75 |  |
|  |  |  |  |  |  |  |  |  |  |  |  |  |  |
| 3-1 | First | 46.7 (14, 6, 8) | 26.7 (8, 4, 4) | 56.7 (17, 12, 5) |  | 6.03 ± 1.80 |  | 6.30 ± 1.53 |  | 59.57 ± 20.16 |  | 63.00 ± 17.51 | * |
|  | Second | 33.3 (10, 5, 5) | 50.0 (15, 4, 11) | 46.7 (14, 5, 9) |  | 5.37 ± 2.24 |  | 6.93 ± 1.06 |  | 55.60 ± 23.31 |  | 72.43 ± 14.20 |  |
|  |  |  |  |  |  |  |  |  |  |  |  |  |  |
| 3-2 | First | 40.0 (12, 7, 5) | 30.0 (9, 4, 5) | 40.0 (12, 7, 5) |  | 6.00 ± 2.07 | * | 6.23 ± 1.48 |  | 61.67 ± 24.74 |  | 64.07 ± 19.63 |  |
|  | Second | 30.0 (9, 4, 5) | 56.7 (17, 9, 8) | 36.7 (11, 5, 6) |  | 4.87 ± 2.23 |  | 6.67 ± 1.22 |  | 52.60 ± 25.35 |  | 68.50 ± 16.82 |  |
|  |  |  |  |  |  |  |  |  |  |  |  |  |  |
| 3-3 | First | 38.8 (31, 14, 17) | 38.8 (31, 17, 14) | 45.0 (36, 21, 15) |  | 5.61 ± 1.97 |  | 6.78 ± 1.17 |  | 54.79 ± 22.60 |  | 68.26 ± 17.18 |  |
|  | Second | 33.8 (27, 17, 10) | 42.5 (34, 17, 17) | 42.5 (34, 19, 15) |  | 5.08 ± 2.12 |  | 6.90 ± 1.35 |  | 52.01 ± 21.70 |  | 67.66 ± 19.10 |  |
|  |  |  |  |  |  |  |  |  |  |  |  |  |  |
| 3-4 | First | 45.0 (18, 8, 10) | 30.0 (12, 5, 7) | 30.0 (12, 6, 6) |  | 5.98 ± 2.06 | ** | 6.80 ± 1.40 |  | 59.43 ± 24.14 | * | 66.43 ± 19.17 |  |
|  | Second | 25.0 (10, 5, 5) | 40.0 (16, 6, 10) | 27.5 (11, 7, 4) |  | 4.68 ± 2.10 |  | 6.48 ± 1.69 |  | 48.68 ± 22.41 |  | 63.43 ± 19.90 |  |
|  |  |  |  |  |  |  |  |  |  |  |  |  |  |

*, *p* < 0.05; **, *p* < 0.01 (paired *t*-test);

^#^, Percentage (Total, TR pattern, RT pattern); ^##^, Average ± SD.

**Supplementary Table S2. Summary of statistical analyses of EEG indices.**

| Exp | EEG analysis^#^ | Meditation^$^ | *α* wave / *β* wave^$^ |
| --- | --- | --- | --- |
| 1 | Exp DIM < Linear DIM | 13 (4 , 9) | 6 (1 , 5) |
|  | Exp DIM > Linear DIM | 10 (6 , 4) | 7 (5 , 2) |
|  | First > Second | 8 (4 , 4) | 3 (1 , 2) |
|  | First < Second | 15 (6 , 9) | 10 (5 , 5) |
|  | no significant | 7 (5 , 2) | 17 (9 , 8) |
| 2-1 | DIM50 > CONST | 9 (2 , 7) | 8 (3 , 5) |
|  | DIM50 < CONST | 13 (9 , 4) | 7 (5 , 2) |
|  | First > Second | 6 (2 , 4) | 5 (3 , 2) |
|  | First < Second | 16 (9 , 7) | 10 (5 , 5) |
|  | no significant | 7 (3 , 4) | 14 (6 , 8) |
| 2-2 | DIM70 > CONST | 9 (4 , 5) | 8 (3 , 5) |
|  | DIM70 < CONST | 9 (5 , 4) | 6 (3 , 3) |
|  | First > Second | 8 (4 , 4) | 6 (3 , 3) |
|  | First < Second | 10 (5 , 5) | 8 (3 , 5) |
|  | no significant | 8 (2 , 6) | 12 (5 , 7) |
| 3-1 | AL0.1 > DIM70 | 14 (7 , 7) | 6 (3 , 3) |
|  | AL0.1 < DIM70 | 5 (3 , 2) | 12 (7 , 5) |
|  | First > Second | 9 (7 , 2) | 8 (3 , 5) |
|  | First < Second | 10 (3 , 7) | 10 (7 , 3) |
|  | no significant | 9 (4 , 5) | 10 (4 , 6) |
| 3-2 | AL0.2 > DIM70 | 14 (5 , 9) | 9 (3 , 6) |
|  | AL0.2 < DIM70 | 12 (8 , 4) | 10 (5 , 5) |
|  | First > Second | 9 (5 , 4) | 8 (3 , 5) |
|  | First < Second | 17 (8 , 9) | 11 (5 , 6) |
|  | no significant | 4 (2 , 2) | 11 (7 , 4) |
| 3-3 | AL1.0 > DIM70 | 27 (12 , 15) | 20 (9 , 11) |
|  | AL1.0 < DIM70 | 34 (20 , 14) | 28 (17 , 11) |
|  | First > Second | 26 (12 , 14) | 20 (9 , 11) |
|  | First < Second | 35 (20 , 15) | 28 (17 , 11) |
|  | no significant | 18 (7 , 11) | 31 (13 , 18) |
| 3-4 | AL1.0 > CONST | 20 (8 , 12) | 16 (10 , 6) |
|  | AL1.0 < CONST | 14 (10 , 4) | 8 (4 , 4) |
|  | First > Second | 12 (8 , 4) | 14 (10 , 4) |
|  | First < Second | 22 (10 , 12) | 10 (4 , 6) |
|  | no significant | 5 (2 , 3) | 15 (6 , 9) |

^#^, *p* < 0.05 (Wilcoxon signed rank test); ^$^, Total (TR pattern, RT pattern).

**Supplementary Table S3. Frequency of perceived change in illuminance (first trial vs second trial).**

| Exp | Target condition Reference condition | Cone adaptation period (0 - 7 min) | | | | | | |  | Rod adaptation period (7 - 15 min) | | | | | | |
| --- | --- | --- | --- | --- | --- | --- | --- | --- | --- | --- | --- | --- | --- | --- | --- | --- |
|  |  | Brightened | | |  | Darkened | | |  | Brightened | | |  | Darkened | | |
|  |  | Freq.$ | n# |  |  | Freq.$ | n# |  |  | Freq.$ | n# |  |  | Freq.$ | n# |  |
|  |  |  |  |  |  |  |  |  |  |  |  |  |  |  |  |  |
| 2-1 | First | 0.63 | 19 (6 , 13) |  |  | 0.68 | 20 (16 , 4) |  |  | 0.47 | 14 (6 , 8) |  |  | 0.30 | 9 (3 , 6) |  |
|  | Second | 0.67 | 20 (14 , 6) |  |  | 0.53 | 16 (5 , 11) |  |  | 0.67 | 20 (15 , 5) |  |  | 0.47 | 14 (8 , 6) |  |
|  |  |  |  |  |  |  |  |  |  |  |  |  |  |  |  |  |
| 2-2 | First | 0.50 | 15 (8 , 7) |  |  | 0.27 | 8 (5 , 3) |  |  | 0.57 | 17 (11 , 6) |  |  | 0.50 | 15 (8 , 7) |  |
|  | Second | 0.70 | 21 (13 , 8) |  |  | 0.27 | 8 (3 , 5) |  |  | 0.60 | 18 (9 , 9) |  |  | 0.50 | 15 (5 , 10) |  |
|  |  |  |  |  |  |  |  |  |  |  |  |  |  |  |  |  |
| 3-1 | First | 0.23 | 7 (4 , 3) |  |  | 0.27 | 8 (5 , 3) |  |  | 0.33 | 10 (4 , 6) |  |  | 0.53 | 16 (14 , 2) |  |
|  | Second | 0.30 | 9 (4 , 5) |  |  | 0.13 | 4 (2 , 2) |  |  | 0.17 | 5 (4 , 1) |  |  | 0.57 | 17 (3 , 14) |  |
|  |  |  |  |  |  |  |  |  |  |  |  |  |  |  |  |  |
| 3-2 | First | 0.37 | 11 (9 , 2) |  |  | 0.43 | 13 (9 , 4) |  |  | 0.33 | 10 (4 , 6) |  |  | 0.30 | 9 (6 , 3) |  |
|  | Second | 0.50 | 15 (10 , 5) |  |  | 0.47 | 14 (5 , 9) |  |  | 0.37 | 11 (9 , 2) |  |  | 0.47 | 14 (4 , 10) |  |
|  |  |  |  |  |  |  |  |  |  |  |  |  |  |  |  |  |
| 3-3 | First | 0.43 | 34 (12 , 22) | * |  | 0.31 | 25 (10 , 15) |  |  | 0.40 | 32 (14 , 18) |  |  | 0.19 | 15 (5 , 10) |  |
|  | Second | 0.26 | 21 (8 , 13) |  |  | 0.26 | 21 (6 , 15) |  |  | 0.39 | 31 (16 , 15) |  |  | 0.15 | 12 (8 , 4) |  |
|  |  |  |  |  |  |  |  |  |  |  |  |  |  |  |  |  |
| 3-4 | First | 0.35 | 14 (3 , 11) |  |  | 0.28 | 11 (9 , 2) |  |  | 0.33 | 13 (7 , 6) |  |  | 0.20 | 8 (4 , 4) |  |
|  | Second | 0.33 | 13 (9 , 4) |  |  | 0.25 | 10 (6 , 4) |  |  | 0.30 | 12 (10 , 2) |  |  | 0.20 | 8 (4 , 4) |  |
|  |  |  |  |  |  |  |  |  |  |  |  |  |  |  |  |  |

^$^, Counts (n) per number of normal subjects;

^#^, Total counts (counts in TR pattern, counts in RT pattern);

*, *p <* 0.05 (Goodness of fit test for the Poisson distribution).

## Supplementary Figures

**Supplementary Fig. S1. Comparisons of *α* wave / *β* wave in EEG.**

Red and black curves indicate the averaged values of *α* wave / *β* wave in EEG under target and reference conditions, respectively.

**Supplementary Fig. S2. Comparisons of meditation scores in EEG.**

Red and black curves indicate the averaged meditation scores in EEG under target and reference conditions, respectively.

**Supplementary Fig. S3. Dark adaptation curve made by a combination of exponential functions for cone and rod adaptations**

Red curve indicates the estimated threshold curve fitted by two exponential functions for cone and rod adaptations. Small black circles indicate a dark adaptation profile after light adaptation, with light intensity of 38,900 photons/cm^2^ (taken from reference 1).

1. Hecht, S., Haig, C. & Chase, A. M. The influence of light adaptation on subsequent dark adaptation of the eye. *J. Gen. Physiol.* **20**, 831–850 (1937).

## Supplementary Methods

## **Questionnaire**

・At which light exposure did you feel the most sleepy?

・At which light exposure did you feel the most relaxing?

・Are there light exposures you felt the illuminance changing? Answer the number of the trial(s).

・In which environment do you think that you will be able to have a comfortable sleep?

・If there are any concerns about the environment during the experiment, please tell us.
